# Gender as a determinant of physical activity levels and mental health of medical students from Poland and Belarus in the context of the COVID-19 pandemic

**DOI:** 10.3389/fpubh.2023.1192068

**Published:** 2023-07-03

**Authors:** Joanna Baj-Korpak, Kamil Zaworski, Ewa Szymczuk, Andrei Shpakou

**Affiliations:** ^1^Department of Physiotherapy, Faculty of Health Sciences, Faculty of Health Sciences, John Paul II University of Applied Sciences in Biala Podlaska, Biala Podlaska, Poland; ^2^Department of Nursing, Faculty of Health Sciences, John Paul II University of Applied Sciences in Biala Podlaska, Biala Podlaska, Poland; ^3^Yanka Kupala State University of Grodno, Grodno, Belarus; ^4^Department of Integrated Medical Care, Faculty of Health Sciences, Medical University of Bialystok, Bialystok, Poland

**Keywords:** physical activity, COVID-19, kinesiophobia, medical students, gender

## Abstract

**Background:**

COVID-19 pandemic has brought about unfavourable changes regarding both physical activity (PA) levels and patterns of behaviour associated with mental health. The study sought to assess PA levels and kinesiophobia in medical students from Poland and Belarus taking account of gender.

**Methods:**

A total of 779 students (405 students from University of Grodno (UG), Belarus, and 374 students from John Paul II University of Applied Sciences (ABNS) in Biala Podlaska, Poland) took part in the study. Women constituted 74.2% of the study population. A diagnostic survey as well as two research tools, i.e., the International Physical Activity Questionnaire (IPAQ)—short form, and the Tampa Scale for Kinesiophobia (TAMPA) were employed in the study.

**Results:**

Students from ABNS manifested significantly higher levels of PA. Taking into account gender, male respondents displayed significantly higher levels of MET-min/week (MET—metabolic equivalent of task). As for kinesiophobia, significantly higher levels were demonstrated by students from UG. Its higher levels were also noted among women.

**Conclusion:**

The findings of the study did not reveal strong correlations between kinesiophobia and PA levels in students from Poland and Belarus in the context of different approaches to the pandemic that both countries adopted. Students from ABNS proved to be more physically active. In turn, participants from UG exhibited significantly higher levels of kinesiophobia. Gender was the factor that significantly differentiated levels of kinesiophobia, with women displaying its higher levels.

## 1. Introduction

Physical activity (PA) is one of the most important and fundamental human needs that contributes to maintaining and improving health. It helps to prevent and treat non-infectious diseases and exerts a positive influence on mental health, quality of life and well-being (1). Properly administered, it can reduce negative effects of ageing processes and help to maintain full physical, mental and social health for as long as possible (2). Unfortunately, some negative changes in the levels of PA and well-being caused by COVID-19 have been noted recently. After the World Health Organization (WHO) had declared the state of pandemic in 2020 (3), a lot of countries (including Poland) imposed restrictions aimed at reducing the spread of SARS-CoV-2. The restrictions forced people to change their PA habits (4).

Similar to other European Union countries, Poland introduced tough restrictions (lockdown) to prevent the spread of the disease. The approach adopted by Belarus was much less restrictive compared to that taken in Poland. Belarus was one of the few Eastern European countries that did not impose any quarantine. Belarus did not adopt a similar antipandemic strategy, denied the presence of the virus and took no action during the first months of the pandemic. The two countries adopted completely different attitudes to the COVID-19 pandemic, which may have affected PA and kinesiophobia levels in the populations.

### 1.1. Pandemic background worldwide and in the two studied countries

COVID-19 was first noted in Wuhan (China) in December 2019. It quickly spread to all parts of the world. In most countries, coping with the virus was based on introducing social distancing. The fact that people were forced to stay at home longer had a negative impact on their PA levels (5, 6). Such lifestyle changes caused by the COVID-19 pandemic may have long-term effects on physical and mental health of the population (7). A lot of individuals affected by the disease experienced various emotional, cognitive and physical disorders. These include stress, anxiety, depression, post-traumatic stress disorders and insomnia (8, 9). COVID-19 may also cause stress in individuals directly exposed to the virus, which may result in chronic anxiety and create financial difficulties (10). The factors that may magnify negative consequences of the pandemic include self-isolation, social distance or quarantine (10–12).

While some pandemic-related stressors affect everyone, many have an impact on women in particular (13). Research shows that women experience higher levels of stress, anxiety, depression and post-traumatic stress disorders (14, 15).

Alsalhe et al. (10) confirm that the COVID-19 pandemic exerts a considerable influence on mental health, which is manifested, among others, by anxiety. Simultaneously, they stress the importance of PA in the process of alleviating anxiety symptoms. More and more evidence points to numerous benefits of taking up PA (16). Moderate-intensity PA improves immunological functions and reduces the risk of viral respiratory infections (17).

### 1.2. Physical activity and mental health

PA seems to be effective when it comes to maintaining or improving physical and mental health particularly during the COVID-19 pandemic. In terms of the population, a number of guidelines and recommendations regarding PA levels have been developed (1, 18, 19). Being physically active brings a lot of benefits to mental health such as preventing the deterioration of cognitive abilities or relieving symptoms of depression and anxiety as well as improving educational achievements. Regular physical activity reduces the risk of cardiovascular diseases, improves muscle and bone mass, reduces pain and fatigue, and improves well-being (20). It also contributes to maintaining proper body mass and fosters well-being (21). The pandemic enhanced the role of providing conditions for and possibilities of being physically active regardless of age, gender, financial status, ethnic origin or PA levels (19). As Global status report on physical activity (19) states, the COVID-19 showed that PA can no longer be perceived as an additional (secondary) element of public policy. The time has come for all countries to formulate a policy that would promote PA as “must have” and to give everyone equal chances of being active (22). Even though it is common knowledge that regular PA improves mental and physical health at every age, currently more than 80% of adolescents and 27% of adults do not meet WHO’s recommendations regarding PA (19). Data show that 1.4 billion adults (27.5% of world adult population) do not reach recommended levels of PA (23). What is disturbing is that no improvement has been observed (24).

Considerable differences in PA levels between regions, countries, age groups and gender can be observed (23). In the majority of countries, women are less active than men. When a slight increase in PA levels in adolescents was noted, it was usually among boys, which enhances gender differences in PA that are lifelong (19).

### 1.3. Physical activity and kinesiophobia

Regular PA performed at a younger age may be a significant motivator for activity at an older age. It can be explained through the continuity theory (25), which states that human beings are inclined to lead the lifestyle as it has been when it comes to leisure time and habits (26). Knapik et al. (27) noted a strong negative correlation between kinesiophobia and habitual physical activity in women aged 60 or older. It points to lower levels of fear of movement in physically active older women.

The issues of physical activity and kinesiophobia, correlations between manifested levels of these variables as well as possible health consequences provided inspiration to carry out the present study.

In the literature of the subject, the term “kinesiophobia” is mainly used to describe an irrational state of fear of movement stemming from previous injuries. A person experiencing it is afraid as they feel susceptible to injuries (28). Anxiety (more or less conscious) accompanies us throughout life, and it is linked to the need of security (29). The term “phobia” is mainly associated with deep anxiety states manifested through psychosomatic symptoms and specific behaviours. In the case of PA, typical phobic symptoms occur quite rarely due to the fact that not taking up PA is treated as avoidance behaviour (30). According to Saulicz et al. (25), we deal with a more or less conscious reaction (“lack of time”, “no immediate results”) or denial (removing the need for activity from one’s awareness).

Kinesiophobia belongs to is a category of avoidance behaviours (31). It should be treated not as fear of pain but as a broader element, i.e., fear of movement-related pain—feeling physical and/or mental discomfort (pain, fatigue, exhaustion, fear of being ridiculed for being unfit, negative perception of particular forms of activity by the society).

It is a manifestation of personality predisposition to physical activity (32). It can be seen as fear of fatigue or exhaustion and, from a psychological standpoint, as fear of being ridiculed for not performing any PA. Socio-demographic factors such as gender may affect kinesiophobia levels (29, 33).

Fear of movement (kinesiophobia) seems to be insufficiently investigated in the context of the COVID-19 pandemic. Most commonly, it is assessed in patients with musculoskeletal pains, spinal pains, degenerative changes in peripheral joints and chronic diseases (34–36). It may contribute greatly to decreasing PA levels and, consequently, to increasing the risk of occurrence of musculoskeletal disorders and chronic diseases (diabetes, cardiovascular diseases) (37). Based on the literature review, Alpalhão et al. (38) noted that kinesiophobia is one of the most important factors determining PA levels.

The study aimed to assess the levels of PA and kinesiophobia in students from two countries in which different approaches to the COVID-19 pandemic were adopted. In Poland, a lot of restrictions were introduced, whereas a completely different approach was favoured in Belarus. Bearing in mind that, from the point of view of mental health, young people (including students) and women may be more susceptible to negative effects of the COVID-19 pandemic (39), the current study sought to assess the levels of PA and kinesiophobia in medical students from Poland and Belarus taking into consideration gender. It involved finding out what (if any) correlations there are between these variables.

## 2. Materials and methods

### 2.1. Participants

The study was carried out on medical students from Poland and Belarus. A purposive sampling method was employed—the number of participants and gender distribution were similar in both groups. A total of 779 respondents (374 students from John Paul II University of Applied Sciences (ABNS) in Biala Podlaska, Poland, and 405 students from University of Grodno (UG), Belarus) took part in the study. In both study groups, women (W) constituted the majority of participants. Taking into consideration the age of the respondents, students from UG were younger (Table 1). It stems from the fact that in Poland, students complete their secondary education and begin higher education at a later age.

**Table 1:** Characteristics of the study population taking account of the respondents’ age (*N* = 779).

Group				Age				Total
				17–20	21–30	31–40	>40	
ABNS	Gender	M	N	19	74	8	4	105
			%	20.0%	35.2%	18.6%	15.4%	28.1%
		W	N	76	136	35	22	269
			%	80.0%	64.8%	81.4%	84.6%	71.9%
	Total		N	95	210	43	26	374
			%	100.0%	100.0%	100.0%	100.0%	100.0%
V Kramer		0.182	12.387^a^	3	**0.006** ^*^	**0.005** ^*^		
UG	Gender	M	N	93	4	0	0	97
			%	24.9%	12.9%	0%	0%	24.0%
		W	N	281	27	0	0	308
			%	75.1%	87.1%	0%	0%	76.0%
	Total		N	374	31	0	0	405
			%	100.0%	100.0%	0%	0%	100.0%
Phi		0.075	2.249^a^	1	0.134	0.188^c^		
Total	Gender	M	N	112	78	8	4	202
			%	23.9%	32.4%	18.6%	15.4%	25.9%
		W	N	357	163	35	22	577
			%	76.1%	67.6%	81.4%	84.6%	74.1%
	Total		N	469	241	43	26	779
			%	100.0%	100.0%	100.0%	100.0%	100.0%
V Kramer		0.107	8.928	3	**0.030** ^*^	**0.030** ^*^		
Coefficient		Value	Chi-squared	*df*	*p*	*p* Monte Carlo		

^*^Statistically significant differences at *p* < 0.05.

### 2.2. Methods

A diagnostic survey as well as three research tools, i.e., the International Physical Activity Questionnaire (IPAQ)—short form, the Tampa Scale for Kinesiophobia (TAMPA) and the authors’ own questionnaire regarding respondents’ socio-demographic data were employed in the study. Due to the issues discussed, research results were analysed with regard to gender.

IPAQ short form consists of seven questions concerning types of PA which constitute elements of everyday life (moderate-to-vigorous PA, walking and time spent sitting). The questions refer to PA performed in the last 7 days. Activities done for at least 10 min at a time are taken into consideration. Total energy expenditure is calculated by multiplying frequency and duration of PA and corresponding intensity expressed in Metabolic Equivalent of Task (MET) units, followed by adding up the obtained results for all activities performed in the last 7 days. MET corresponds to an energy expenditure at rest with an oxygen uptake of 3.5 mL/min/kg of body mass. Three categories (levels) of PA were distinguished, i.e., high, moderate and low (40). In this study, the Cronbach’s alpha coefficient for IPAQ was 0.74.

TAMPA was developed by Kori et al. in order to assess fear of movement-related pain or (re)injury (41). This questionnaire consists of 17 items, where each item is assessed on a four-point Likert scale. The total score of the scale ranges from 17 to 68, and scores above 37 point to a high degree of kinesiophobia (42). Internal consistency assessed with Cronbach’s alpha was 0.72.

### 2.3. Study design

The participants’ socio-demographic data as well as data on variables of PA were gathered. The respondents were informed about anonymity of data collection. All the participants gave their informed consent to take part in the study. The study was carried out in April and May 2022. The survey questionnaires, i.e., IPAQ, TAMPA and the authors’ questionnaire on socio-demographic data (paper version), were completed by the respondents at their place of study, with one of the researchers present. The Bioethics Committee of the ABNS in Biala Podlaska approved the study protocol (Resolution no. 4/2022). This study was conducted within the project ‘Physical activity and mental health of medical students from Poland and Belarus in the context of the dynamically changing situation of the COVID-19 pandemic’ funded by the National Agency for Academic Exchange (NAWA). The usefulness of the research as well as project outline had been presented in an earlier publication (43).

### 2.4. Statistical analysis

The data collected were analysed using SPSS 17.0 (Softonic, Ashburn, VA, United States). Quantitative variables were presented taking into account mean $\bar{x}$ , median, standard deviation (SD), ranges and 95% confidence intervals. Categorical variables were presented as percentage and the number of units from the same group.

To calculate qualitative data, correlation coefficients based on the chi-squared test, i.e., Phi and V Kramer, were used. Comparative analysis between groups ABNS and UG was performed using the *t*-test for independent samples. In order to determine effect size, weighted standard deviation was used as a denominator in the case of Cohen’s *d*. As for Hedges’ *g*, summary standard deviation together with correction coefficient were used, whereas for Glass’s delta, standard deviation of the control group was applied. Correlations between qualitative variables were calculated using Spearman’s rho, which measures the strength and direction of correlations between variables. The coefficient values always range from −1 to 1, and the strength of correlations was classified as follows: 0 to 0.3—weak correlation, 0.3 to 0.5—moderate correlation, and 0.5 to 1—strong/very strong correlation (44). Statistical significance was set at *p* < 0.05. The Cronbach’s alpha coefficient was used to measure internal consistency of the results. The coefficient values range from 0 to 1, and the higher the value (closer to 1), the greater the reliability of the scale. In psychometric studies, it is assumed that results above 0.7 point to satisfactory reliability of the scale.

## 3. Results

Men (M) constituted 25.8% of all the study participants (*N* = 779). This proportion reflects a tendency that women dominate when it comes to attending medical courses in both countries (Table 1). Taking into account gender, significant differences were noted in the whole study population as well as in respondents from ABNS.

The first step to achieve the aim of the study was to determine PA levels in students from Poland and Belarus.

The data presented in Table 2 show that students from ABNS were considerably more active, and the difference was significant. Taking into account gender, significantly higher values of MET-min/week were found in men.

**Table 2:** Differences in MET-min/week taking account of gender and the place of study (*t*-test).

Variable		*N*	$\bar{x}$ (±SD)	*t*	*df*	*p*	Quotient *F* variances	*p* variances
University	ABNS	374	4777.2 (±5197.3)	5.0720	777	0.001^***^	5.2918	0.001^***^
	UG	405	3339.6 (±2259.3)					
Gender	W	577	3710.5 (±3443.7)	3.7838	777	0.0002^***^	2.2999	0.001^***^
	M	202	4941.9 (±5222.5)					
W	ABNS	308	4281.2 (±4387.5)	3.7628	575	0.0002^***^	3.9202	0.001^***^
	UG	269	3212.0 (±2216.0)					
M	ABNS	97	6047.9 (±6708.8)	3.2029	200.0	0.0016^**^	8.0944	0.001^***^
	UG	105	3744.7 (±2358.1)					
ABNS	W	269	4281.2 (±6708.8)	2.9852	372	0.003^**^	2.3381	0.001^***^
	M	105	6047.9 (±6708.8)					
UG	W	308	3212.0 (±2216.0)	2.0327	403	0.0427^*^	1.1324	0.4310
	M	97	3744.7 (±2358.1)					

^*^Statistically significant differences at *p* < 0.05. ^**^Statistically significant differences at *p* < 0.005. ^***^Statistically significant differences at *p* < 0.001.

**Figure 1:**
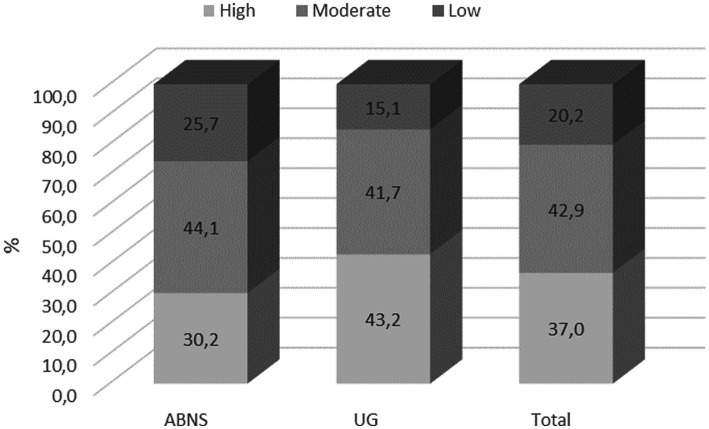
Percentage distribution of the respondents’ PA levels (IPAQ).

Students who met the criteria of IPAQ for the moderate level of PA constituted the largest proportion of the study population, while high levels of PA were manifested by a third of the respondents. MET-min/week median was 2970.0, which points to considerable involvement of students in broadly understood physical culture. Taking into consideration the place of study, it was noted that students from Poland were more active than their peers from Belarus (Me 3,246 MET-min/week and 2,880 MET-min/week, respectively). However, more students from UG met the criteria of high levels of PA. Detailed data are included in Table 3 and Figure 1.

**Table 3:** Respondents’ PA levels (IPAQ—MET-min/week).

University	PA levels	*N*	$\bar{x}$ (±SD)	Min	Max	*Q*25	Median	*Q*75
Total	High	288	6023.1 (±4718.2)	1537.0	42,768	3190.0	4783.7	7087.8
	Moderate	334	3651.4 (±3244.1)	495.0	31,560	1546.0	2790.0	4358.0
	Low	157	1178.4 (±1066.9)	0.0	6,984	396.0	996.0	1596.0
	Total	**779**	**4029.8 (±4014.7)**	**0.0**	**42,768**	**1530.0**	**2970.0**	**5010.0**
ABNS	High	113	8298.1 (±6284.7)	1584.0	42,768	4551.0	6612.0	10,506
	Moderate	165	4472.2 (±4153.5)	495.0	31,560	1752.0	3492.0	5,832
	Low	96	1157.1 (±1249.9)	0.0	6,984	204.8	822.0	1,638
	Total	**374**	**8298.1 (±5197.3)**	**0.0**	**42,768**	**1386.0**	**3246.0**	**6,426**
UG	High	175	4554.0 (±2400.7)	1537.0	14,398	2777.0	4110.0	5695.4
	Moderate	169	2850.1 (±1646.8)	587.0	11,962	1535.0	2666.0	3770.0
	Low	61	1211.9 (±695.5)	113.0	3,558	763.4	1130.0	1530.0
	Total	**405**	**3339.6 (±2259.3)**	**113.0**	**14,398**	**1676.0**	**2880.0**	**4434.0**

Bold value indicates total PA.

**Figure 2:**
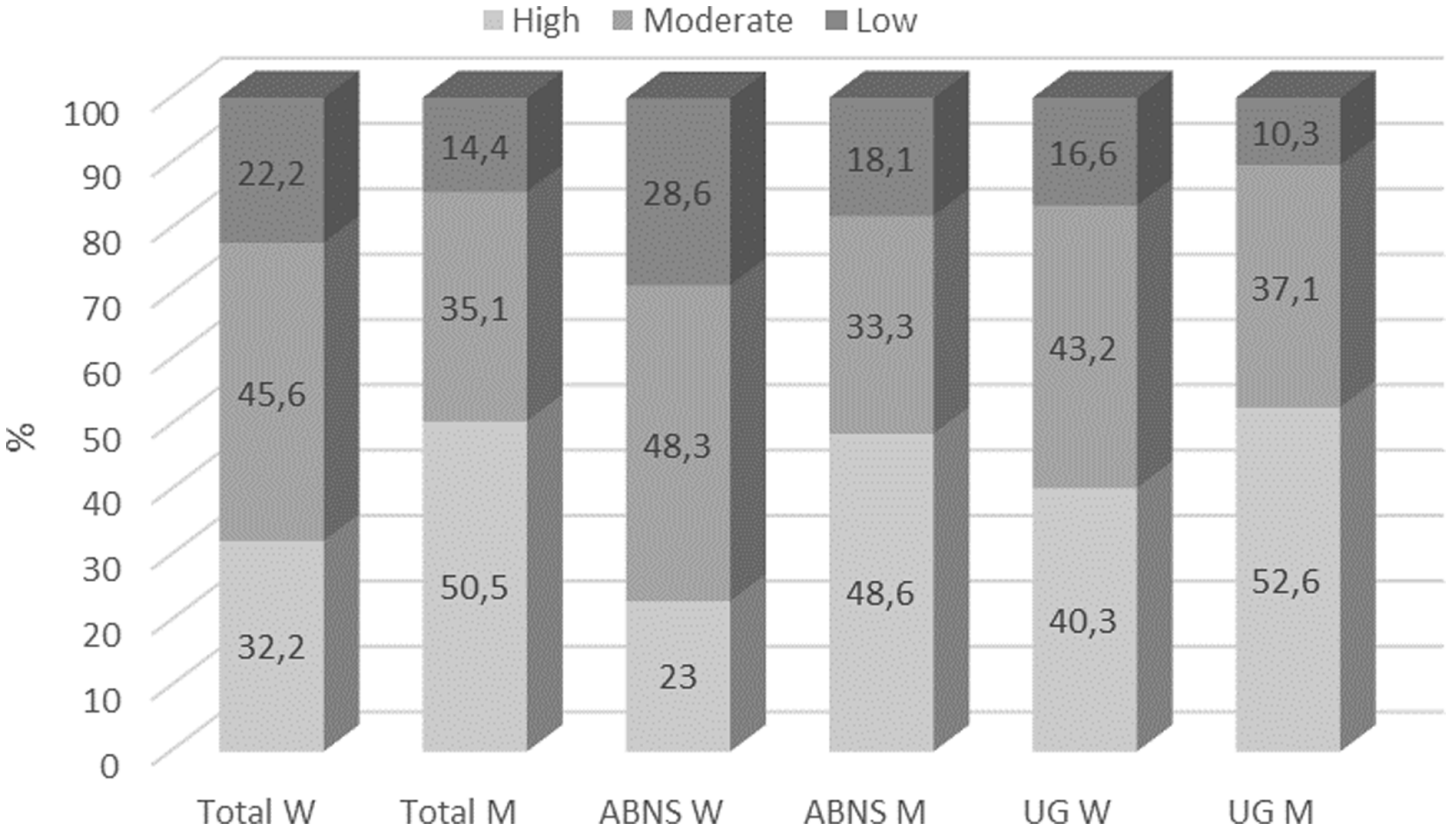
Percentage distribution of the respondents’ PA levels taking account of gender (IPAQ).

Assuming that gender may determine PA levels, results presented in Figure 2 show that a larger proportion of men met the IPAQ criteria for the highest level of PA—more than half of the male respondents demonstrated high PA levels. This correlation was observed in students from both universities (Figure 2).

During the COVID-19 pandemic, kinesiophobia was significantly greater among students from UG (the index value >37 indicates its high level). Higher index values were also noted among women, and the difference was significant. Male and female students from ABNS did not manifest high levels of kinesiophobia (Table 4).

**Table 4:** Differences in the levels of kinesiophobia taking account of the place of study and gender.

University	*N*	$\bar{x}$ (±SD)	Min	Max	*Q*25	Median	*Q*75	*t*	*df*	*p*	Quotient *F*	*p* variances
ABNS	374	34.70 (±5.72)	19.00	52.00	30.00	35.00	39.00	10.16	777	0.001^***^	1.141	0.197
UG	405	39.02 (±6.11)	23.00	61.00	35.00	39.00	43.00					
W	577	37.25 (±6.23)	19.00	61.00	33.00	38.00	41.00	−2.25	777	0.024^*^	1.074	0.526
M	202	36.09 (±6.45)	23.00	56.00	31.00	36.00	40.00					
ABNS W	269	34.99 (±5.80)	23.00	49.00	30.00	33.00	37.00	−1.55	372	0.122	1.129	0.477
ABNS M	105	33.97 (±5.46)	19.00	52.00	30.00	35.00	39.00					
UG W	308	39.22 (±5.91)	23.0	56.0	34.0	39.0	42.0	−1.18	403	0.238	1.276	0.125
UG M	97	38.38 (±6.68)	23.0	56.0	34.0	39.0	42.0					
Total	779	36.95 (±6.30)	19.00	61.00	32.00	38.00	41.00					

^*^Statistically significant differences at *p* < 0.05. ^***^Statistically significant differences at *p* < 0.001.

The next stage of the analysis involved determining the level of kinesiophobia in students manifesting certain levels of PA (established according to IPAQ methodology). Considering the cut-off point of 37 pts. (with mean index values), it was noted that the respondents demonstrating moderate levels of PA simultaneously displayed high levels of kinesiophobia (TAMPA<37 pts.). Taking median into account, the comparison revealed high index values in all groups (PA levels) as well as in PA as a whole. In each case, the cut-off point was exceeded. In the case of gender, it was observed that fear of movement-related pain was greater in women—both for the whole study population and with regard to particular universities. However, it is worth noting that the value of the kinesiophobia index in female students from ABNS was below 37 pts.. The respondents from Belarus manifested high levels of fear of movement (Table 5).

**Table 5:** The levels of kinesiophobia taking account of PA levels and gender.

	PA levels	*N*	$\bar{x}$ (±SD)	Min	Max	*Q*25	Median	*Q*75
Total	High	288	36.66 (±6.24)	20.00	56.00	32.00	37.00	41.00
	Moderate	334	37.37 (±6.47)	19.00	61.00	32.00	38.00	41.00
	Low	157	36.57 (±6.03)	23.00	49.00	33.00	37.00	40.00
	Total	779	36.95 (±6.30)	19.00	61.00	32.00	38.00	41.00
W	High	186	37.19 (±6.04)	20.00	55.00	33.00	38.00	41.00
	Moderate	263	37.53 (±6.56)	19.00	61.00	32.00	38.00	42.00
	Low	128	36.75 (±5.80)	23.00	49.00	33.00	37.00	40.00
	Total	577	37.25 (±6.23)	19.00	61.00	33.00	38.00	41.00
M	High	102	35.69 (±6.16)	23.00	56.00	31.00	35.00	40.00
	Moderate	71	36.80 (±6.15)	23.00	56.00	31.00	37.00	41.00
	Low	29	35.76 (±7.00)	23.00	49.00	30.00	36.00	42.00
	Total	202	36.09 (±6.45)	23.00	56.00	31.00	36.00	40.00
ABNS W	High	62.0	34.97 (±5.03)	20.00	52.00	28.00	36.00	40.00
	Moderate	130.0	34.98 (±5.03)	19.00	49.00	30.00	35.00	39.00
	Low	77.0	35.01 (±5.03)	23.00	47.00	31.00	35.00	39.00
	Total	269.0	34.99 (±5.03)	19.00	52.00	30.00	35.00	39.00
ABNS M	High	51	33.39 (±5.03)	25.00	45.00	29.00	33.00	37.00
	Moderate	35	35.46 (±5.86)	23.00	49.00	31.00	36.00	39.00
	Low	19	32.79 (±5.52)	23.00	45.00	30.00	31.00	37.00
	Total	105	33.97 (±5.46)	23.00	49.00	30.00	33.00	37.00
UG K	High^*^	124	38.31 (±5.40)	26.00	55.00	35.00	38.50	42.00
	Moderate^*^	133	40.02 (±6.39)	27.00	61.00	37.00	39.00	44.00
	Low	51	39.37 (±5.59)	28.00	49.00	35.00	39.00	44.00
	Total	308	39.22 (±5.91)	26.00	61.00	36.00	39.00	43.00
UG M	High	51	37.98 (±7.06)	23.00	56.00	32.00	39.00	43.00
	Moderate	36	38.11 (±6.22)	28.00	56.00	33.50	39.50	41.50
	Low	10	41.40 (±6.11)	29.00	49.00	37.00	42.50	46.00
	Total	97	38.38 (±6.68)	23.00	56.00	34.00	39.00	42.00

^*^Statistically significant differences at *p* < 0.05 between TAMPA values for high and moderate levels in group UG W.

Data presented in Table 6 indicate that for the whole study population, gender significantly differentiated both kinesiophobia and PA levels. As for the place of study, in the groups of students both from Poland and Belarus, significant differences between women and men were only noted in MET-min/week. Higher PA levels were observed in men.

**Table 6:** Differences in kinesiophobia levels (TAMPA) and MET-min/week (IPAQ) taking account of gender—correlations (Spearman’s rho).

University	Questionnaire	Gender	*N*	$\bar{x}$ (±SD)	Standard error of means	*t*-test for mean value equality							Correlations
						*t*	*df*	*p*	Difference in means	Standard error of dffferences	95% confidence interval for differences in means		
											Lower	Upper	
Total	TAMPA	M	202	36.09 (±6.45)	0.45	−2.26	777.00	0.024*	−1.16	0.51	−2.17	−0.15	−0.07
		W	577	37.25 (±6.23)	0.26								
	IPAQ	M	202	4941.91 (±5222.48)	367.45	3.12	264.71	**0.002** ^*^	1231.40	394.43	454.78	2008.02	
		W	577	3710.51 (±3443.67)	143.36								
ABNS	TAMPA	M	105	33.97 (±5.46)	0.53	−1.55	372.00	0.121	−1.02	0.66	−2.31	0.27	0.002
		W	269	34.99 (±5.81)	0.35								
	IPAQ	M	105	6047.92 (±6708.81)	654.71	2.50	140.11	**0.014** ^*^	1766.67	707.26	368.40	3164.95	
		W	269	4281.25 (±4387.50)	267.51								
UG	TAMPA	M	97	38.38 (±6.68)	0.68	−1.18	403.00	0.238	−0.84	0.71	−2.24	0.56	−0.12
		W	308	39.22 (±5.91)	0.34								
	IPAQ	M	97	3744.68 (±2358.06)	239.42	2.03	403.00	**0.043** ^*^	532.64	262.04	17.50	1047.78	
		W	308	3212.04 (±2215.96)	126.27								

^*^Statistically significant differences at *p* < 0.05.

In none of the analysed areas (groups) were strong correlations between kinesiophobia levels and MET-min/week found (Table 6). Table 7 shows data defining the strength of correlations between these indices. Both for the whole study population and for participants from ABNS and UG, small effect size was revealed.

**Table 7:** Effect size for independent samples—kinesiophobia levels (TAMPA) and MET-min/week (IPAQ).

University	Questionnaire	Coefficient	Standardiser	Score estimation^*^	Confidence interval 95%	
					Lower	Upper
Total	TAMPA	Cohen’s *d*	6.288	−0.185	−0.345	−0.024
		Hedges’ *g*	6.294	−0.184	−0.345	−0.024
		Glass’s delta	6.230	−0.186	−0.347	−0.026
	IPAQ	Cohen’s *d*	3980.78445	0.309	0.148	0.470
		Hedges’ *g*	3984.63206	0.309	0.148	0.470
		Glass’s delta	3443.67139	0.358	0.196	0.519
ABNS	TAMPA	Cohen’s *d*	5.716	−0.179	−0.404	0.047
		Hedges’ *g*	5.727	−0.178	−0.404	0.047
		Glass’s delta	5.812	−0.176	−0.402	0.050
	IPAQ	Cohen’s *d*	5143.08336	0.344	0.116	0.570
		Hedges’ *g*	5153.48159	0.343	0.116	0.569
		Glass’s delta	4387.49711	0.403	0.174	0.630
UG	TAMPA	Cohen’s *d*	6.105	−0.137	−0.366	0.091
		Hedges’ *g*	6.117	−0.137	−0.365	0.091
		Glass’s delta	5.914	−0.142	−0.370	0.087
	IPAQ	Cohen’s *d*	2250.62521	0.237	0.008	0.465
		Hedges’ *g*	2254.82458	0.236	0.008	0.464
		Glass’s delta	2215.96223	0.240	0.011	0.469

^*^0.2—small effect, 0.5—medium effect, and 0.8 and above—large effect.

## 4. Discussion

In the current study, an attempt was made at assessing PA levels as well as behaviours related to mental health (kinesiophobia) in two countries that adopted different approaches to the pandemic. When planning our research, we assumed that apart from different systemic activities, gender may constitute a variable determining health-related behaviours regarding physical (in)activity. The findings of our study showed that men displayed higher PA levels during the pandemic. Taking into account different approaches to the pandemic in the countries included in the study, we noted higher values of MET-min/week and lower levels of kinesiophobia in students from Poland.

The COVID-19 pandemic was the time of changes in everyday activities of students. During lockdown, young people spent most of the time at home and they were mainly inactive. They did not socialise enough; they did not have enough social interactions and opportunities to move around freely, which meant more sedentary behaviours (45). According to Huang et al. (46), young people are at a higher risk of suffering from mental disorders compared to other age groups.

Women are more susceptible to stress and, consequently, some disorders such as anxiety and depression (47, 48). It was confirmed in the case of medical students, where women exhibited higher levels of emotional exhaustion. They also manifested lower perceptions of physical and psychological quality of life as well as higher dispositions for emphatic concern and anxiety (49). It is in line with the findings of the present study, which prove that women demonstrate higher levels of fear of movement-related pain. It is also confirmed by Kluszczyńska et al. (32). They found higher levels of kinesiophobia in female participants compared to their male counterparts. The correlation between kinesiophobia and gender was also observed by Kocjan and Knapik (50). Women demonstrated higher levels of kinesiophobia in the psychological domain.

Qiu et al. (11) attempted to measure psychological distress (anxiety, concern, despair) in the general population of China during the COVID-19 pandemic. They stated that women were more prone to stress and more likely to develop post-traumatic stress disorder.

Due to the fact that PA is a significant element of our lifestyle and it forms the basis for all rehabilitation procedures, the issue of kinesiophobia should not only be thoroughly investigated in terms of theoretical knowledge but also recognised when it comes to particular social groups, situations or patients. There is still a scarcity of data on kinesiophobia in the context of the COVID-19 pandemic. Therefore, we decided to conduct an investigation on the group that was affected significantly by the pandemic (distance learning, limited socialising). Kinesiophobia may develop in individuals who do not have conditions that would prevent them from taking up PA also during the pandemic (51). Our study focused on medical students, i.e., young people who possess knowledge regarding health promotion and prevention. An interesting thing is that the highest levels of kinesiophobia were found in moderately active individuals (with the exception of UG M).

Kinesiophobia levels in our study participants were linked to the time spent performing PA per week. High levels of kinesiophobia mainly observed in students from Belarus may stem from the fact that the investigation was carried out during the prolonged period of the COVID-19 pandemic and that individuals who had not been very active beforehand were also included in the analysis. Similar conclusions were also drawn by other researchers (51–54). Furthermore, the fact that the virus mutated into a more severe variant (55) may have contributed to higher levels of kinesiophobia in our study participants.

In the study on patients who were infected with SARS-CoV-2, Bahar Özdemir (56) noted a significant correlation between kinesiophobia and low back pain. This observation may indicate that pain increases fear of movement, and thus people become less active, which may constitute a vicious circle. Restrictions introduced during the pandemic (e.g., distance learning) forced our study participants to exhibit sedentary behaviours, which may have led to musculoskeletal pains.

In our study we analysed correlations between kinesiophobia and declared PA levels in students from Poland and Belarus in the context of different approaches to the pandemic adopted in these countries. The study did not show strong correlations between these variables, both in general and in particular groups (countries).

Barğı et al. (57) noted a positive linear correlation between insufficient PA levels and sedentary behaviours in the course of the pandemic. Interesting conclusions were reached by Bertrand et al. (58), who found that during the COVID-19 pandemic, women manifested higher levels of PA. They attributed it to higher motivation among women (peer pressure, weight loss or its maintenance). During the pandemic, a lot of people (men in particular), despite quarantine and restrictions, began to take up various forms of PA in order to strengthen their immune system, improve their mental health and reduce negative psychological effects of the pandemic (59). Bajramovic et al. (60) showed that women manifested lower levels of PA compared to men, which is consistent with our findings. However, the reported PA had a positive effect on students’ well-being both among women and men (60). Despite pandemic-related restrictions, the vast majority of students from Poland and Belarus met WHO’s recommendations regarding PA (1) Rousset et al. (61) drew similar conclusions with regard to young adult students from France. In the current study, IPAQ showed that respondents from both Biala Podlaska and Grodno manifested moderate and high levels of PA. Students from ABNS displayed significantly higher levels of PA in comparison to their peers from UG. Taking gender into account, significantly higher values of MET-min/week were noted among men. Similar observations were made by Orlandi et al. (62), who claimed that women demonstrated lower levels of PA before lockdown but they did not have a tendency to reduce PA levels during the pandemic to such an extent as men did. The findings of our study, which was carried out when the pandemic was being ‘suppressed’, point to higher levels of PA among men from both countries.

The introduction of drastic measures caused by the COVID-19 pandemic aimed to prevent the spread of SARS-CoV-2. However, it also produced negative effects such as mental health issues and post-traumatic stress disorder symptoms, avoiding other people, anger, anxiety, frustration, boredom, stigmatisation or financial losses (39, 54, 63, 64) as well as an increase in negative health consequences and routine behaviours like reduced PA (walking, exercising) and increased sedentary behaviours (65, 66). Negative effects of lockdown are linked with the development of unhealthy everyday habits in the population of students manifested by, e.g., decreasing levels of PA. These consequences should not be ignored when it comes to the health of the population and young people in particular. We agree with Saulicz et al. (25), who stated that insufficient participation in PA at a later stage of life may be linked to high levels of kinesiophobia that appeared during adolescence. Reduced PA during youth produces high levels of kinesiophobia at later stages. Sedentary behaviours that are mainly a consequence of pandemic restrictions may become a permanent element of lifestyle. Research indicates that the COVID-19 pandemic may have a negative influence on PA (67). Restrictions introduced during the pandemic encourage people to stay at home, which increases the amount of time spent sitting and enhances sedentary behaviours. Should similar critical situations occur, special attention must be paid to anti-pandemic policies adopted by governments (closing schools or universities, limiting possibilities of realising sports and recreational programmes, etc.) in order to avoid negative effects not only on physical but also on mental health.

With regard to results presented in the current work as well the findings of the study conducted by Saulicz et al. (68), kinesiophobia is a feature inseparably connected with PA. As for the self-determination theory, high levels of kinesiophobia may create an emotional barrier determining inner motivation to perform PA (68). Interactions between low levels of PA and high levels of kinesiophobia constitute a barrier to getting involved in PA, thus forming the aforementioned “vicious circle”.

### 4.1. Limitation

Like all scientific investigations, our study also has some limitations. One of them is the determination of socio-economic status of the participants. Due to the fact that the study involved students from Grodno and Biala Podlaska, it is difficult to generalise the findings to the whole population in both countries (Belarus and Poland) or to representatives of both genders. Still, we are convinced that they point to a certain trend.

Another limitation of the study is its retrospective and cross-sectional character. The respondents completed the questionnaires at one point in time only, which may have increased the probability of not remembering earlier situations in detail.

Identifying kinesiophobia and dealing with it by taking an individual multidisciplinary approach may lead to an improvement in the quality of life. The present study aimed to assess kinesiophobia and PA in medical students taking account of gender and thereby to provide a better insight into correlations between these variables.

Moreover, it should be stressed that the study did not include peaks of the COVID-19 pandemic in Poland and in Belarus as the aim was to determine the levels of PA and kinesiophobia in the context of the pandemic (effects of the COVID-19 pandemic). A longitudinal study conducted over a longer period of time might have provided more thorough data. However, due to the unpredictability and high dynamics of the spread of SARS-CoV-2, carrying out such research within the project was not possible (final stage of the COVID-19 pandemic).

## 5. Conclusion

Because of different approaches to the pandemic adopted in Poland and in Belarus, the participation of the students in broadly understood physical culture also differed. Students from Poland proved to be more physically active, which was confirmed by higher values of MET-min/week. In turn, study participants from Belarus manifested significantly higher levels of kinesiophobia. Gender was the factor that significantly differentiated levels of kinesiophobia, with women demonstrating its higher levels.

In the population under study, no significant correlations between kinesiophobia and PA levels taking account of gender were revealed, with the exception of women from UG, who displayed high and moderate levels of PA.

## Data availability statement

The data analyzed in this study is subject to the following licenses/restrictions: The data analysed in this article are a database extract from a study carried out under the projket—NAWA Intervention Grants Program (BPN/GIN/2021/1/00084/U/00001). Requests to access these datasets should be directed to JB-K, email: j.baj-korpak@dyd.akademiabialska.pl.

## Ethics statement

The studies involving human participants were conducted in accordance with the Declaration of Helsinki. They were reviewed and approved by Bioethics Committee of the ABNS in Biala Podlaska (protocol code 4/2022 18.05.2022). The patients/participants provided their written informed consent to participate in this study.

## Author contributions

JB-K, KZ, and AS: conceptualisation, methodology, formal analysis, and writing—review and editing. JB-K, KZ, ES, and AS: investigation and data curation. JB-K and KZ: writing—original draft preparation. JB-K: supervision. All authors contributed to the article and approved the submitted version.

## Funding

This research was funded by the National Agency for Academic Exchange (NAWA) under the NAWA Intervention Grants Program (BPN/GIN/2021/1/00084/U/00001).

## Conflict of interest

The authors declare that the research was conducted in the absence of any commercial or financial relationships that could be construed as a potential conflict of interest.

## Publisher’s note

All claims expressed in this article are solely those of the authors and do not necessarily represent those of their affiliated organizations, or those of the publisher, the editors and the reviewers. Any product that may be evaluated in this article, or claim that may be made by its manufacturer, is not guaranteed or endorsed by the publisher.
